# Plant expression and characterization of the transmission-blocking vaccine candidate *Pf*GAP50

**DOI:** 10.1186/s12896-015-0225-x

**Published:** 2015-12-01

**Authors:** Veronique Beiss, Holger Spiegel, Alexander Boes, Matthias Scheuermayer, Andreas Reimann, Stefan Schillberg, Rainer Fischer

**Affiliations:** Fraunhofer Institute for Molecular Biology and Applied Ecology (IME), Forckenbeckstrasse 6, 52074 Aachen, Germany; Research Center for Infectious Diseases, University of Wuerzburg, Josef Schneider Str. 2/Bau D15, 97080 Wuerzburg, Germany; RWTH Aachen University, Institute for Molecular Biotechnology, Worringer Weg 1, 52074 Aachen, Germany

**Keywords:** *Plasmodium falciparum*, Sexual stage, Gametes, Agroinfiltration, Plant-made vaccines, Plastid targeting

## Abstract

**Background:**

Despite the limited success after decades of intensive research and development efforts, vaccination still represents the most promising strategy to significantly reduce the disease burden in malaria endemic regions. Besides the ultimate goal of inducing sterile protection in vaccinated individuals, the prevention of transmission by so-called transmission blocking vaccines (TBVs) is being regarded as an important feature of an efficient malaria eradication strategy. Recently, *Plasmodium falciparum* GAP50 (*Pf*GAP50), a 44.6 kDa transmembrane protein that forms an essential part of the invasion machinery (glideosome) multi-protein complex, has been proposed as novel potential transmission-blocking candidate. Plant-based expression systems combine the advantages of eukaryotic expression with a up-scaling potential and a good product safety profile suitable for vaccine production. In this study we investigated the feasibility to use the transient plant expression to produce *Pf*GAP50 suitable for the induction of parasite specific inhibitory antibodies.

**Results:**

We performed the transient expression of recombinant *Pf*GAP50 in *Nicotiana benthamiana* leaves using endoplasmatic reticulum (ER) and plastid targeting. After IMAC-purification the protein yield and integrity was investigated by SDS-PAGE and Western Blot. Rabbit immune IgG derived by the immunization with the plastid-targeted variant of *Pf*GAP50 was analyzed by immune fluorescence assay (IFA) and zygote inhibition assay (ZIA). *Pf*GAP50 could be produced in both subcellular compartments at different yields IMAC (Immobilized Metal Affinity Chromatography) purification from extract yielded up to 4.1 μg/g recombinant protein per fresh leaf material for ER-retarded and16.2 μg/g recombinant protein per fresh leave material for plasmid targeted *Pf*GAP50, respectively. IgG from rabbit sera generated by immunization with the recombinant protein specifically recognized different parasite stages in immunofluorescence assay. Furthermore up to 55 % inhibition in an *in vitro* zygote inhibition assay could be achieved using *Pf*GAP50-specific rabbit immune IgG.

**Conclusions:**

The results of this study demonstrate that the plant-produced *Pf*GAP50 is functional regarding the presentation of inhibitory epitopes and could be considered as component of a transmission-blocking malaria vaccine formulation.

**Electronic supplementary material:**

The online version of this article (doi:10.1186/s12896-015-0225-x) contains supplementary material, which is available to authorized users.

## Background

Still affecting millions of people around the world, predominantly in developing countries [1], malaria is one of the most relevant poverty-related infectious tropical diseases. Even though prophylactic and therapeutic agents exists, they are not broadly available and affordable for the local populations. Measures of vector elimination and control by draining mosquito breeding pools, application of insecticides, and bed nets have not been sufficiently effective in many cases. Additionally, increasing resistances of the parasite and the vector against both, chemical control measures and medical treatments are a growing problem [2, 3]. Therefore, the availability of a vaccine is being regarded as an essential component of a successful malaria eradication strategy [4]. Despite intense research efforts no vaccine that provides robust sterile protection against malaria is available today. Involving two different hosts, mosquito and man, the life cycle of *P. falciparum* is complex and features three different stages. As summarized in the WHO rainbow table [5], the majority of proteins used in the context of malaria vaccine candidates today and in the past, are either pre-erythrocytic, or blood stage antigens like *Pf*CSP [6], proteins from the merozoite surface protein family (*Pf*MSPn) [7], *Pf*AMA1 [8] and other blood stage surface proteins [9]. After successful completion of clinical trials GSK’s circumsporozoite protein (CSP)-based pre-erythrocytic stage vaccine Mosquirix® [10] received a positive opinion from EMA and is expected to enter the market soon, following WHO recommendation and clearance by respective national regulators. Even though being by far the most advanced malaria vaccine, the impact of Mosquirix® is expected to be limited by moderate efficacy (only up to 60–70 % of the vaccinees protected) and relatively short-lived protection [11]. Besides vaccines targeting the pre-erythrocytic stage of the parasite and therefore aiming at the induction of sterile protection by preventing the initial establishment of the infection, there are at least two other types of vaccines. Blood stage vaccines could be useful to suppress manifestation of clinical symptoms caused by high parasite load in the blood of the patients, while so called transmission blocking vaccinestarget the sexual stages of the parasite to prevent proliferation in, or trafficking through the mosquito and thereby inhibit the transmission of the parasites from infected to healthy individuals. In the context of malaria eradication efforts and after a recent update of the Malaria Vaccine Technology Roadmap in 2013 [12], transmission-blocking vaccines have recently received elevated attention. The first and most advanced transmission-blocking malaria vaccine candidate is *Pfs*25, a 24 kDa post-fertilization macrogamete/zygote surface antigen featuring four epidermal growth factor (EGF)-like domains [13, 14]. It has been shown in different studies that immunization with recombinant *Pfs*25 induces antibodies with strong transmission-blocking activity [15–18]. Being exclusively expressed in parasite stages that develop after fertilization within the mosquito and thereby being naturally not exposed to the human immune system the highly conserved *Pfs*25 represents an excellent target for vaccine induced transmission-blocking antibody responses. Other potentially transmission-blocking vaccine candidates in development are the pre-fertilization gamete/gametocyte antigens *Pfs*230 and *Pfs*48/45. In contrast to *Pfs*25 these proteins have been shown to be targets of natural immune responses [19] since they are already expressed on gametocytes that (within infected erythrocytes) occur in the human host.

*Pf*GAP50, a 44.6 kDa transmembrane protein, forms an essential part of the actin-myosin motor complex driven invasion machinery (glideosome) associated to the multi-protein complex called inner membrane complex (IMC) [20–23]. Detailed investigations on the role of *Pf*GAP50 during sexual stage development presented by Simon *et al.* [24] indicate that the protein relocates from the IMC to the plasma membrane during gametocyte activation and gametocyte egress from erythrocytes triggered by change of temperature, pH and presence of xanthurenic acid encountered within the mosquito midgut after a blood meal. Since in this work it has also been shown that *Pf*GAP50 may protect the gametes from complement-mediated lysis by binding the human complement Factor H, the protein can be regarded as novel transmission-blocking candidate.

Plant-based transient expression systems are robust, fast and scalable platforms capable of oxidative folding, assembly of multimeric proteins and high level expression [25]. Several pharmaceutically relevant proteins like monoclonal antibodies [26], therapeutic enzymes [27] as well as antigens, including malaria vaccine candidates [28–33] have been produced successfully by transient plant expression. The *Agrobacterium tumefaciens* transient plant expression system offers the chance to compare the efficiency of recombinant protein expression in various subcellular compartments (ER, cytoplasm, plastids) with different features regarding oxidative folding or post-translational modifications [34, 35].

Here, we report the successful plant-based production and characterization of the novel TBV candidate *Pf*GAP50. The plastid targeted *Pf*GAP50 was obtained at high quality by one-step IMAC purification and used for the immunization of rabbits. The resulting rabbit immune IgG preparations were used in different *in vitro* assays to confirm the induction of antibodies that recognize *Pf*GAP50 in the native context.

## Results

### Cloning the *Pf*GAP50 expression constructs

**Fig. 1:**
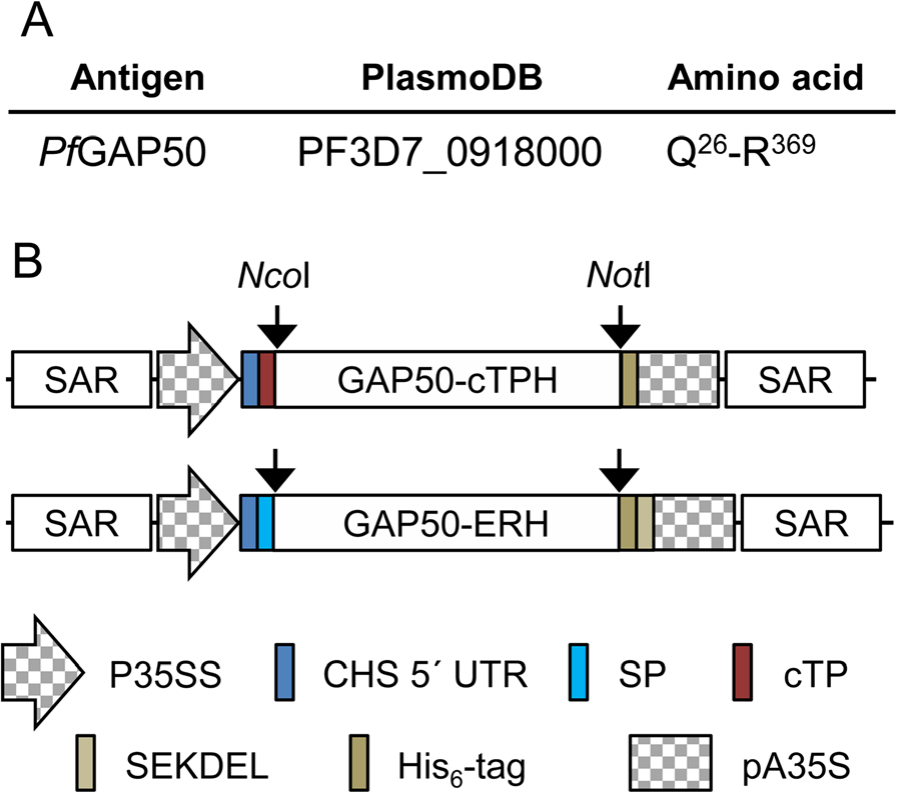
Gene IDs and plant expression cassettes. **a** Names, accession numbers and amino acid sequence range of *Pf*GAP50 (for further details refer to the methods section). **b** Schematic drawing of the expression cassettes in the plant binary expression vector pTRAkc (not to scale). SAR: scaffold attachment region; P35SS: *Cauliflower mosaic virus* 35S promoter with duplicated 35S enhancer region; CHS 5ʹ UTR: 5ʹ untranslated region of the *Petroselinum crispum* chalcone synthase gene; SP: transit peptide sequence of the murine antibody 24 heavy chain [60]; TP: chloroplast targeting signal from small subunit of RuBisCO from *Solanum tuberosum*; GAP50: *Pf*GAP50; His_6_-tag: six histidine tag for IMAC purification; SEKDEL: ER-retention signal. pA35S: *Cauliflower mosaic virus* 35S polyadenylation signal. Relevant restriction sites are indicated

The cDNA coding for the extracellular domain of *Pf*GAP50 (Fig. 1a) was cloned into the binary plant expression vectors pTRAkc-ERH and pTRAkc-cTPH. For the cloning we used cDNA encoding amino acids Q26 to R369 featuring the *Pf*GAP50 without the native N-terminal signal peptide (to be replaced by the plant-specific targeting signals) and without the transmembrane domain to enhance the solubilty of the protein targeted to either the ER or plastid. The resulting pTRAkc-GAP50-ERH (Fig. 1b) construct features sequences for an N-terminal signal peptide (for targeting the protein to the secretory pathway), and a C-terminal His6-tag (for affinity purification and detection) followed by a SEKDEL ER-retrieval signal (for ER-retention). The second expression construct pTRAkc-GAP50-cTPH (Fig. 1b) provides an N-terminal chloroplast targeting peptide and a C-terminal His6-tag.

### Transient expression and purification of *Pf*GAP50 in *N. benthamiana*

**Fig. 2:**
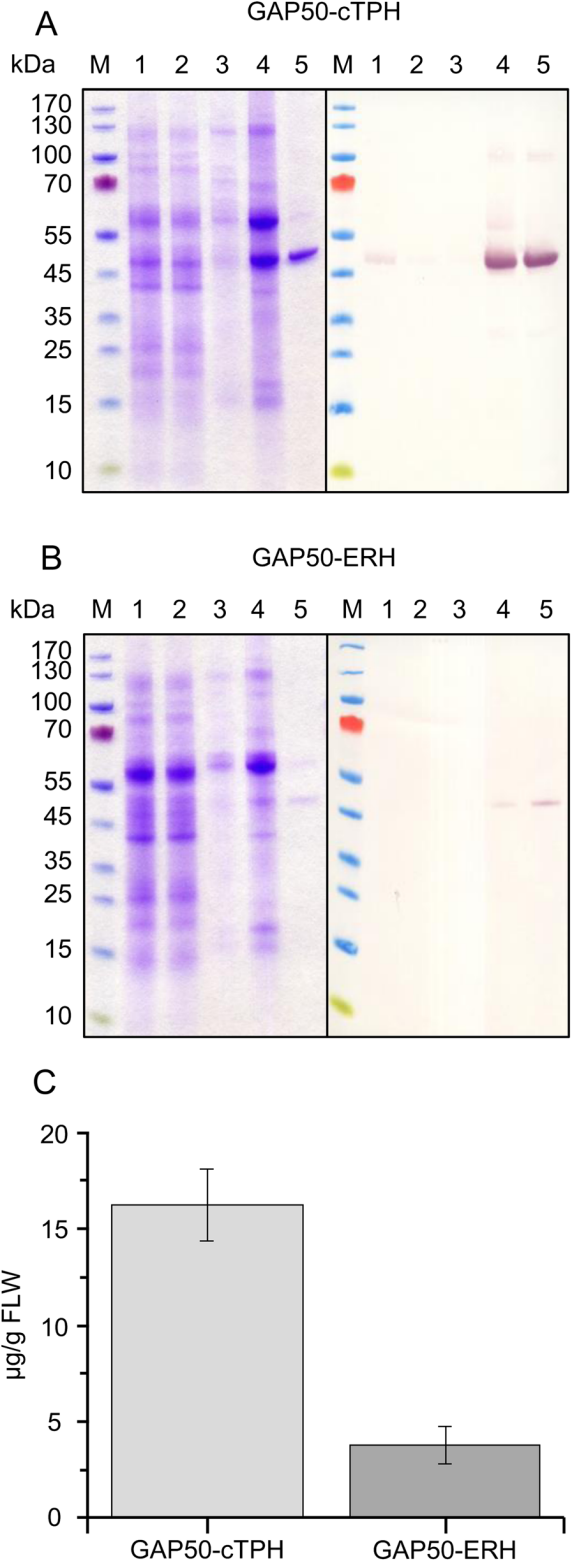
SDS-PAGE/immunoblot analysis of plant produced *Pf*GAP50. **a**: Purification of plastid-targeted *Pf*GAP50 (*Pf*GAP50-cTPH). For purification, 7.0 g infiltrated leaf material were used. Reducing SDS-PAGE (left panel) and immunoblot (right panel). **b**: Purification of ER-retarded *Pf*GAP50 (*Pf*GAP50-ERH). For purification, 4.5 g infiltrated leaf material were used. Reducing SDS-PAGE (left panel) and immunoblot (right panel). M: Prestained protein marker (Page Ruler Fermentas), 1: 3 μL load (plant extract), 2: 3 μL flow-through, 3: 6 μL elution step 1 (10 mM imidazole), 4: 6 μL elution step 2 (100 mM imidazole), 5: 6 μL elution step 3 (250 mM imidazole). Western blot was detected with rabbit anti-His6 serum and alkaline phosphatase labeled goat anti rabbit serum. **c**: Plot of mean values and standard deviation of the yields for finally purified (E3) *Pf*GAP50-cTPH and *Pf*GAP50-ERH, determined by densitometric analysis (against BSA equivalents) of SDS-PAGE from three independent replicates (SDS-PAGE shown in additional file 1)

Both expression constructs were used for transient plant expression of *Pf*GAP50. The recombinant protein was purified by IMAC from plant extracts prepared from infiltrated *N. benthamiana* leaves 5 days post-infiltration (dpi). The experiment was repeated independently three times for both constructs and a mock purification using wild type material was carried out once to simplify the identification of potential contaminants. Figure 2a and b exemplarily show the SDS-PAGE and immunoblot analysis of one experiment per construct (SDS-PAGE featuring the samples from all replicates is shown in additional file 1). The simple one-step purification procedure using three step elution (10 mM, 100 mM and 250 mM) yielded *Pf*GAP50 proteins at different levels of purity and high integrity. As shown in Fig. 2a and b, elution at 10 mM Imidazole only yielded few host cell proteins (to a large proportion presumably the large subunit of Ribulose-1,5-bisphosphat-carboxylase/-oxygenase (RuBisCo), 56 kDa) and no detectable *Pf*GAP50-ERH or *Pf*GAP50-cTPH (as shown by His_6_-specific immunoblot), at 100 nM we detected host cell proteins at higher abundance as well as detectable amounts of target proteins. The elution fraction E3 (250 mM Imidazole) in both cases contained the highest amount of *Pf*GAP50 and one major host cell protein band running at the size of the large subunit of RuBisCo. Yields were determined by densitometric analysis of SDS-PAGE lanes containing the E3 fractions against a BSA standard curve derived from 4 different concentrations of BSA. The clear differences in yield between the ER-retarded and the chloroplast-targeted variant of the protein were observed in several other experiments (data not shown). Figure 2c shows the average E3 yields obtained after three expression and purification experiments of *Pf*GAP50-cTPH (16.2 ± 1.8 μg/g fresh leaf weight (FLW), 7 g leaf material for each repeat) and *Pf*GAP50-ERH (4.1 ± 0.98 μg/g FLW, 4.5 g leaf material for each repeat). The differences are statistically significant *P =* 0.0005. The higher accumulation levels of intact *Pf*GAP50 also correlated with the achieved relative abundance calculated by densitometric analysis of SDS-PAGE image (Additional file 1) for each purification experiment. For both *Pf*GAP50 variants (*Pf*GAP50-cTPH and *Pf*GAP50-ERH) the highest purity was obtained in the third elution step (E3 fraction) at 250 mM imidazole, while the previous step at 100 mM imidazole still contained a number of host cell proteins, presumably the large subunit of the RuBisCo together with the recombinant protein. This protein was also present as the major contamination in the E3 fraction. In E3 *Pf*GAP50-cTPH was obtained at a relative abundance of 73 % ± 8.5 while *Pf*GAP50-ERH was less pure (32.3 % ± 3.1). The corresponding E3 fraction from an up-scaled infiltration experiment (*Pf*GAP50-cTPH) was used for the immunization of rabbits as described in the methods section.

### Determination of antigen-specific titers

**Fig. 3:**
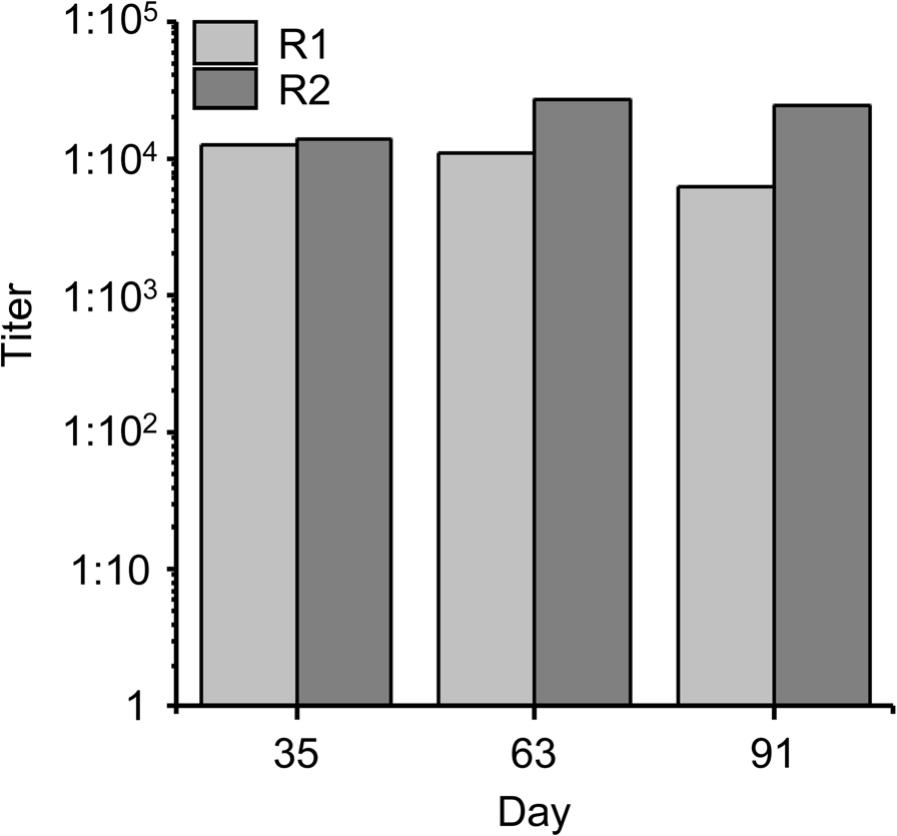
*Pf*GAP50-specific titers in rabbit immune sera. Immune sera were analyzed by direct ELISA after the first (day 35), the second (day 63) and third boost (day 91). The threshold for titer definition was twofold the background signal obtained from the pre-immune sera. R1: rabbit 1, R2: rabbit 2

To initially assess the immunogenicity and parasite inhibitory efficacy of the recombinant *Pf*GAP50-cTPH, two rabbits were immunized using a hyper immunization protocol. Endpoint IgG titers against *Pf*GAP50 were determined in serum samples taken on days 0, 35, 63 and 91 as described in the methods section. As shown in Fig. 3, the resulting titers are moderate for both rabbits with a maximum antibody titer observed after day 63 with 1:2.6 x 10^4^ for rabbit number two. The titers did not increase after subsequent boosts.

### Immunofluorescence assay

**Fig. 4:**
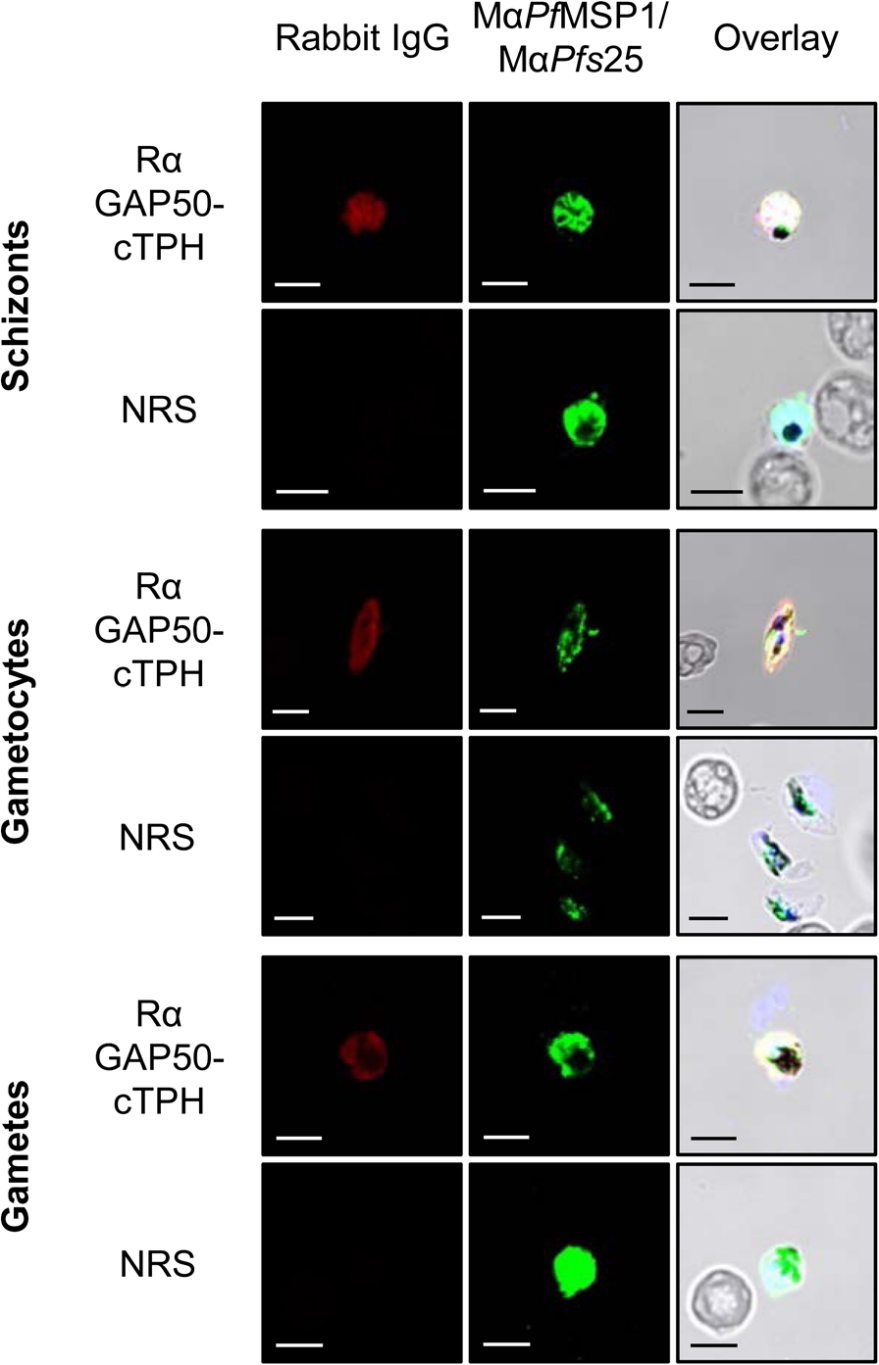
Indirect immunofluorescence assays demonstrating the reactivity of *Pf*GAP50 specific rabbit immune IgG against different stage *P. falciparum* parasites. IFAs were performed on schizonts, gametocytes and gametes, using *Pf*GAP50 specific rabbit immune IgG or IgG purified from neutral rabbit serum (NRS) as a negative control. Mouse anti-*Pfs*25 and anti-*Pf*MSP1 antibodies were used to co-label the sexual-stage (*Pfs*25) and blood stage (*Pf*MSP1) parasites, respectively. Mouse antibodies were visualized with Alexa Fluor 488-conjugated secondary antibodies (green) and rabbit antibodies with Alexa Fluor 594-conjugated secondary antibodies (red). Bar = 5 μm

An IFA was used to determine the reactivity of *Pf*GAP50-specific rabbit immune IgG against *P. falciparum* schizonts, gametocytes and gametes. As shown in Fig. 4, rabbit antibodies raised against *Pf*GAP50 specifically labeled the surfaces of the three different stages. Co-labeling with mouse antibodies against *Pf*MSP1 and *Pfs*25 was used for stage-specific counterstaining. No fluorescence signal was observed when NRS was used for immunolabeling in control experiments.

### Zygote inhibition assay (ZIA)

**Fig. 5:**
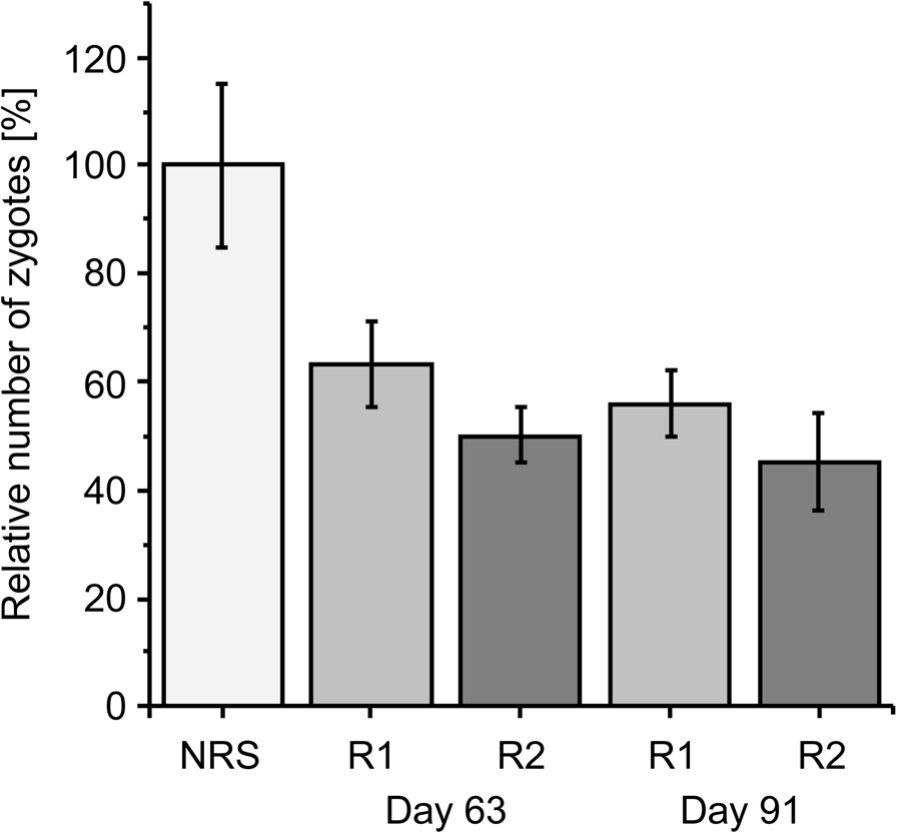
Zygote inhibition assay (ZIA) with *Pf*GAP50 specific rabbit immune IgG. The transmission-blocking potential of the *Pf*GAP50-cTPH specific rabbit immune IgG (rabbit 1 (R1) and rabbit 2 (R2)) from 2^nd^ (day 63) and 3^rd^ bleed (day 91) was assessed in a ZIA experiment in comparison with IgG purified from neutral rabbit serum (NRS) as a negative control. Inhibition was calculated as the reciprocal value of zygote numbers in comparison with the negative control. Error bars were derived by generating mean and SD of the values obtained for immune IgG from the two individual rabbits using data from three technical replicates

The ability of *Pf*GAP50-specific rabbit immune IgG to inhibit zygote development was measured using a ZIA. As shown in Fig. 5, we observed up to 55 % zygote inhibition activity when using 1 mg/mL of the purified rabbit IgG (bleed2, day 63 and bleed 3, day 91 from both rabbits, R1 and R2, respectively).

## Discussion

Mosquirix (RTS’S), a pre-erythrocytic malaria vaccine candidate based on *Pf*CSP presented on the surface of Hepatitis B virus-like particles is now approaching final regulatory approval. Anyhow, data from the respective clinical trials indicate that this vaccine will not match the efficacy and the sustainability of vaccines against other infectious diseases and suggests further efforts towards improved malaria vaccine formulations eventually featuring additional antigens from the blood and the sexual stages of the parasite to introduce functionalities that complement the strategy of inducing sterile protection by immune responses against pre-erythrocytic antigens. In this context a vaccine component that provides transmission blocking activity is being regarded as a valuable contribution to the goal of malaria eradication.

Here, we describe the transient plant-based production and characterization of *Pf*GAP50 as transmission-blocking vaccine candidate antigen. We have used the *A. tumefaciens*-based transient plant expression platform successfully in previous studies for the expression and characterization of different *P. falciparum* antigens from the pre-erythrocytic, the blood and the sexual stage as single proteins [28] or as fusions [33, 36]. Along this line, and driven by the overall vision of implementing this platform for the rapid and cost efficient production of vaccine antigens in the context of malaria vaccine R&D and finally even clinical material, we were interested to evaluate the feasibility to use the transient plant-based expression to produce *Pf*GAP50 suitable for the induction of parasite-specific inhibitory antibodies.

Bosch et al. [37] have produced folded, soluble *Pf*GAP50 in the cytosol of *E. coli* for structural studies. These results indicate that *Pf*GAP50 may not depend on an oxidative environment like the plant endoplasmatic reticulum (ER) for correct folding. Therefore, making use of the possibility to easily address different subcellular compartments within the plants, we generated two expression constructs featuring *Pf*GAP50, one ER-retarded and the other one targeted to the plastids. Even though plastids lack oxidative conditions and the complex, chaperon-based folding machinery of the secretory pathway constituted by the ER and the Golgi apparatus, high-level expression of recombinant proteins has been achieved in plastids for several examples with transient, targeting-based [35, 38, 39] and stable, transplastomic approaches [40].

In our study we observed significantly higher yields for recombinant *Pf*GAP50 (±16.2 ± 1.9 μg/g FLW) targeted to the plastids when compared to *Pf*GAP50 (±4.1 ± 0.98 μg/g FLW) accumulated in the ER. These values were calculated by quantification of the finalmaterial (partially purified by IMAC) and related to yield after extraction and purification. The differences in achieved purities (73 % ± 8.5 for *Pf*GAP50-cTPH and 32.3 % ± 3.1 for *Pf*GAP50-ERH) resulted from a constant amount (around 1-2 μg/g FWL) of a predominant co-purified host cell protein (presumably the large subunit of RuBisCo).

The observation that a 4-fold higher yield could be achieved by chloroplast targeting compared to the ER-retarded version of *Pf*GAP50 was not predictable, but indicates that this strategy should be generally considered when using plant expression systems for the production of recombinant proteins at optimal yields. In a study on the transient and stable plant-based expression of different HIV-I antigen constructs Meyers et al. [41] also compared ER-retardation with chloroplast targeting and found large protein-specific differences in yields, including up to 20-fold higher (HIV-I p17/p24) or 4-fold lower yields (HIV-I p24), by chloroplast targeting compared to ER-retardation. These results also emphasize the versatility of the transient plant expression system as a tool in vaccine candidate development and evaluation, since different properties and requirements of heterologous proteins like vaccine candidate antigens can be addressed by simply testing the respective proteins in the context of different subcellular targeting options.

The yields of 16 and 4 μg/g FLW were calculated for comparative purposes only considering the *Pf*GAP50 amounts found in the elution fraction E3. Since it is obvious that also the fraction E2 contains relevant amounts of *Pf*GAP50 (especially for *Pf*GAP50-cTPH, where E2 seems to contains at least the same amount as E3) it can be speculated that by developing a more advanced extraction and purification strategy [42, 43] it should be possible to significantly increase the yield of *Pf*GAP50-cTPH towards levels of 30-50 μg/g FLW. These assumptions can be used to briefly asses the question of economic perspectives of the production of this transmission-blocking vaccine candidate by transient expression in plants. As basis for this estimation one can refer to the numbers provided by Tusé et al. [44] in an article about the manufacturing economics of plant-made biologics. In this context the per/dose production costs (upstream) for a recombinant butyrylcholinesterase manufactured by transient plant-based expression for therapeutic purposes were calculated as 474 $ (production costs upstream) per dose based on the following variables (Facility capacity: 25 kg recombinant protein/year at expression levels of 500 μg/g, Overall Yield: 100 μg/g FLW, 1 dose: 400 mg). Adapting this calculation to a 25 μg dose, the per/dose production costs for *Pf*GAP50 at an overall yield of 50 μg/g would be 0.06 $ or 0.19 $ at an overall yield (as observed in this study under pre-process development conditions) of 16 μg/g at a capacity of 32 000 000–100 000 000 doses per year. These numbers can be combined with previously calculated numbers presented by [45] assuming that downstream costs will typically account for around 80 % of the total production costs. For the *Pf*GAP50 scenario this would mean between 0.30 $ (yield 50 μg/g) and 0.95 $ (yield 16 μg/g). With these total production costs it should be possible to match a vaccine price that would provide cost effectiveness in a malaria endemic region, given a sufficient efficacy of the vaccine. Based on models [46] and empirical data [47] the cost effectiveness of a pre-erythrocytic vaccine (for the model) and more specifically RTS,S (within a trial setting) has been calculated. These complex calculations do rely on a large number of factors, including efficacy, transmission rate, and look at the socio-economic impact of mild, as well as severe infections and therefore should be regarded as a very rough estimate, when being transferred to another scenario. Anyhow, together with the results from another modeling approach that also involves transmission blocking vaccines [48] it seems reasonable to assume cost effectiveness of a decent transmission blocking vaccine at a vaccine price somewhere between 2 and 10 $, and therefore roughly 10-times higher than the assumed total production costs for *Pf*GAP50 derived from a calculation that was working with a production facility able to produce 32 000 000 to 100 000 000 doses per year.

Because of the better yields and higher purity, the plastid-targeted *Pf*GAP50-cTPH was chosen for rabbit immunization studies. Using a standard hyper immunization protocol moderate titers below 1:1x10^5^were observed in both rabbits. In contrast to other rabbit immunizations we conducted with plant-produced *P. falciparum* antigens under the same conditions [28, 36], in this study we did not observe an increase of antigen-specific titers after the second and/or the third boost. In contrast, for rabbit R1 the titers even slightly decreased over the course of the immunization period. There are different possible explanations for this somehow unexpected result (e.g. lack of suitable T-helper epitopes, immune suppressive activity, influences of animal housing conditions) but given the low number of animals (two) used in this study the observation should probably not be overestimated.

The ability of *Pf*GAP50-cTPH-specific rabbit IgG to recognize *Pf*GAP50 in its native context was assessed by immunofluorescence assays. In these experiments, a staining of blood stage (schizonts) as well as sexual stage parasites (gametocytes and gametes) was observed. Because in schizonts and gametocytes before activation the *Pf*GAP50 is part of the actin-myosin motor complex driven invasion machinery (glideosome) associated to the multi-protein complex called inner membrane complex (IMC), the cell membranes were permeabilized by saponin washing upon preparation of these samples. These results correlate well with what has been observed by Simon et al. [24] after mouse immunization studies performed with recombinant *Pf*GAP50-GST fusions produced in *E. coli* and confirm that properly folded *Pf*GAP50 could be produced by transient plant-based expression.

To initially investigate the parasite inhibitory and thereby transmission-blocking potential of the *Pf*GAP50-cTPH-specific antibodies a zygote inhibition assay was performed by the addition of *Pf*GAP50-cTPH-specific rabbit immune IgG to gametocyte cultures. The observed reduction of zygote numbers after 16 h of incubation between 37 % and 55 % is in good accordance with the 44 % reduction observed by Simon et al. [24] with *Pf*GAP50-specific murine antibodies, providing another indication that the plant-produced *Pf*GAP50 is functional regarding the presentation of inhibitory epitopes and is generally suitable as component of a transmission-blocking malaria vaccine formulation.

Taken together the results encourage the investigation of chloroplast targeting for other malaria vaccine constructs using the transient expression system. This would be especially interesting for antigens like *Pfs*25, *Pf*AMA1, *Pf*MSP3 or others that contain potential N-linked glycosylation sites which are not post-translationally modified in the native context because *Plasmodium* lacks the molecular machinery for N-linked glycosylation [49]. In most cases when potentially N-glycosylated *P.falciparum* antigens are produced in eukaryotic expression hosts N-glycosylation is being prevented by mutation of the NxT/S motifs in the amino acid sequence of the proteins [50]. Another option that has been applied for the generation of diversity covering variants of *Pf*AMA1 [51] is the identification of allelic variations that do not contain the motive at a respective site, allowing to use a native *P.falciparum* sequence instead of an artificial one resulting from the introduced mutation of the N or the T/S residue. Using chloroplast targeting, it is possible to express the fully native, non-glycosylated protein sequence in an environment that is suitable for the expression of at least some folded proteins as already shown for the expression of the disulfide-rich *P.falciparum* sexual stage vaccine antigens *Pfs*25 and *Pfs*28 in the chloroplasts of the eukaryotic green microalgae, *Chlamydomonas reinhardtii* by Gregory et al. [52]*.* Another advantage of chloroplast targeting is the option to attach the recombinant antigens to starch granules by fusion to the granule bound starch synthase (GBSS), which enables the presentation of the antigen in the context of starch granules offering alternative purification strategies as well as potentially improved immunogenicity by the particulate format. This concept has been successfully proven with *Plasmodium berghei* and *Plasmodium falciparum* antigens expressed in a starch granule bound format in *C. reinhardtii by* Dauvillée et al. [53] and also the high immunogenicity of RTS,S the clinically most advanced malaria vaccine candidate based on the presentation of the pre-erythrocytic antigen *Pf*CSP on the surface of hepatitis B virus S antigen (HBs Ag) based virus like particles, suggests the presentation of malaria antigens in a particulate format. In this context it should be also mentioned that plants also offer the possibility to express virus like particles from plant virus coat protein fusion proteins [54].

## Conclusion

The results of this study demonstrate that *Pf*GAP50 can be successfully produced in plants by transient transfection, and that significantly higher levels can be obtained by chloroplast targeting. The plant-produced *Pf*GAP50 is functional regarding the presentation of inhibitory epitopes, can be used to induce parasite inhibitory antibodies and therefore should be considered as an interesting component of a transmission-blocking malaria vaccine formulation. Based on these promising results, we will focus on further optimizing the protein expression and purification as well as on strategies to improve the immunogenicity of the recombinant protein or its formulation before proceeding towards more detailed studies involving more animals and additional, more detailed, functional characterization of the protein itself as well as corresponding immune IgG in different parasite inhibition assays.

## Methods

### Bacteria, plants and parasites

*Agrobacterium tumefaciens* strain GV3101 : : pMP90RK [GmR, KmR, RifR] [55] and *Nicotiana benthamiana* plants were used for the production of the recombinant protein by agroinfiltration. Freshly prepared *P. falciparum* parasites strain NF54 were used for immunofluorescence assay (IFA) and zygote inhibition assay (ZIA) procedures. *P. falciparum* parasites were cultivated as previously described [56].

### Plant expression constructs

The cDNAs encoding the *P. falciparum* strain 3D7 *Pf*GAP50 extracellular domain without the signal peptide and GPI-anchor sequence was obtained as synthetic gene, codon-optimized for *N. benthamiana* from GeneArt (LifeTechnologies, Darmstadt, Germany). The *Pf*GAP50 sequence was introduced via *Nco*I/*Not*I cloning either into the binary vector pTRAkc-ERH [57] between the signal peptide sequence and a His_6_-tag followed by the SEKDEL signal for endoplasmic reticulum (ER) retention [58] or into pTRAkc-cTPH, a modified variant of the binary vector pTRAk-(rbcs)cTP [59] between a plastid targeting signal sequence and a His_6_-tag (H). The resulting plasmids were named pTRAkc-GAP50-cTPH and pTRAkc-GAP50-ERH. All cloning steps were verified by DNA sequencing.

### Transient expression in *N. benthamiana*

The pTRAkc-GAP50-cTPH and pTRAkc-GAP50-ERH vector were introduced into *A. tumefaciens* by electroporation using a Multiporator (Eppendorf AG, Hamburg, Germany) according to the manufacturer’s instructions. Recombinant *A. tumefaciens* carrying pTRAkc-GAP50-cTPH or pTRAkc-GAP50-ERH were used for the transient expression of *Pf*GAP50 either as ER-retarded or as chloroplast targeted variant. Detailed cultivation and infiltration procedures have been reported elsewhere [28, 31, 33].

### Extraction of total soluble protein from *N. benthamiana* leaves

The infiltrated *N. benthamiana* leaves were harvested 5 days post infiltration (dpi) and total soluble protein was extracted as previously described [33].

### Immobilized metal affinity chromatography purification (IMAC)

After pH adjustment, the clarified extract was loaded onto a disposable column filled with 1 ml of Ni^2+^-charged Chelating Sepharose (GE Healthcare, Solingen, Germany). After washing with PBS (10 column volumes), bound proteins were eluted in a three-step gradient using PBS containing 10 mM, 100 mM and 250 mM imidazole.

### SDS-PAGE and immunoblot analysis

SDS-PAGE and immunoblot analysis was performed as previously described [33]. The *Pf*GAP50 variants (*Pf*GAP50-ERH: ER-retarded and *Pf*GAP50-cTPH chloroplast-targeted) were detected with rabbit anti-His_6_ antiserum (GenScript, Piscataway, NY) and an alkaline phosphatase-conjugated goat anti-rabbit secondary antibody (Jackson ImmunoResearch Europe Ltd., Suffolk, UK) both diluted 1:5000 in PBS. BSA standard (NEB) at 900, 600, 300 and 150 ng was used for comparative densitometric quantification of purified *Pf*GAP50. The evaluation was performed using AIDA software (Raytest, Straubenhardt, Germany).

### Rabbit immunization, titer determination and IgG purification

Rabbits were housed, immunized and sampled by Biogenes GmbH (Berlin, Germany), according to national animal welfare regulations. The animal facilities and protocols were reviewed and approved by: Landesamt für Landwirtschaft, Lebensmittelsicherheit und Fischerei MecklenburgVorpommern (LALLF M-V) (Approval No: 7221.3-2-030-13). To isolate the blood after immunization according to national regulations the animals were anesthetized using Ventranquil, stunned using a captive bolt device and exsanguinated by throat cut. Two rabbits were hyper immunized intramuscularly with the plant-derived and purified *Pf*GAP50-cTPH formulated with a proprietary Biogenes adjuvant (an oil in water emulsion containing lipopolysaccharides) on days 0, 7, 14, 28, 49 and 77. 200 μg of antigen were used for prime and 100 μg for the five consecutive boosts. Serum samples were taken on days 35, 63 and 91. For titer determination, the samples from the three bleeds, as well as pre-immune serum, were tested for reactivity with the plant-derived *Pf*GAP50-cTPH by enzyme-linked immunosorbent assay. Titer determination and IgG purification by Protein A was performed as previously described [28]. End-point titers were determined as the highest dilution that gave the double the value of the background (day 0, pre-immune serum).

### Immunofluorescence assay (IFA)

IFAs were carried out on three different *P. falciparum* stages (schizonts, mature gametocytes, and macrogametes) by immunolabeling with 10 μg/mL purified *Pf*GAP50-cTPH-specific rabbit IgG (day 63) or 10 μg/mL IgG purified from normal rabbit serum as previously described [33]. The sexual stages were co-labeled with antibodies against *Pf*Msp1 (schizonts) or *Pfs*25 (mature gametocytes and macrogametes).

### Zygote inhibition assay (ZIA)

The zygote inhibition potential of the *Pf*GAP50-cTPH antisera was investigated by zygote inhibition assay (ZIA) as described in detail by *Simon et al.* [24]. *Pf*GAP50-cTPH-specific rabbit immune IgG purified from day 63 and day 91 were used at concentrations of 1 mg/mL in the final assay volume. The numbers of zygotes were counted in triplicate using a hemocytometer.

## Additional file

Additional file 1:
**SDS-PAGE based quantification (densitometric against a BSA standard) of** ***Pf*** **GAP50 variants in IMAC elution fractions (three independent replicate each).** Mock purification of wt *N. benthamiana* extract was included. Proteins were eluted from the IMAC column using three elution steps (elution 1 (E1): 10 mM imidazole; elution 2 (E2): 100 mM imidazole and elution 3 (E3): 250 mM imidazole). 6 μl of each sample was loaded under reducing conditions. 1: 150 ng BSA/slot; 2: 300 ng BSA/slot; 3: 600 ng BSA/slot; 4: 900 ng BSA/slot; 5–7: E1-3 from mock purification; 8–10: E1-3 from *Pf*GAP50-ERH repeat 1; 11–13: E1-3 from *Pf*GAP50-ERH repeat 2; 14–16: E1-3 from *Pf*GAP50-ERH repeat 3; 17–19: E1-3 from *Pf*GAP50-cTPH repeat 1; 20–22: E1-3 from *Pf*GAP50-cTPH repeat 2; 23–25: E1-3 from *Pf*GAP50-cTPH repeat 3. (PDF 156 kb)

## Abbreviations

*Pf*GAP50: *Plasmodium falciparum* glideosome associated protein 50
TBV: Transmission-blocking vaccine
ERH: ER-retention sequence with His6-tag
cTPH: Plastid targeting sequence with His6-tag
IMC: Inner membrane complex
IFA: Immunofluorescence assay
TBA: Transmission-blocking assay
IMAC: Immobilized metal affinity chromatography
FLW: Fresh leaf weight
P35SS: *Cauliflower mosaic virus* 35S promoter with duplicated 35S enhancer region
CHS 5ʹ UTR: 5ʹ untranslated region of the *Petroselinum crispum* chalcone synthase gene
SP: Transit peptide sequence of the murine antibody 24 heavy chain [60]
TP: Chloroplast targeting signal from small subunit of RuBiSCO from *Solanum tuberosum*
His_6_-tag: Six histidine tag for IMAC purification
SEKDEL: ER-retention signal
pA35S: *Cauliflower mosaic virus* 35S polyadenylation signal
NRS: Neutral rabbit serum
