# Supplementary material for: Plant expression and characterization of the transmission-blocking vaccine candidate PfGAP50
Source: BMC Biotechnol. 2015 Dec 1;15:108. doi: 10.1186/s12896-015-0225-x (PMC4665938; doi:10.1186/s12896-015-0225-x)
Supplement: Additional file 1: — SDS-PAGE based quantification (densitometric against a BSA standard) of Pf GAP50 variants in IMAC elution fractions (three independent replicate each). Mock purification of wt N. benthamiana extract was included. Proteins were eluted from the IMAC column using three elution steps (elution 1 (E1): 10 mM imidazole; elution 2 (E2): 100 mM imidazole and elution 3 (E3): 250 mM imidazole). 6 μl of each sample was loaded under reducing conditions. 1: 150 ng BSA/slot; 2: 300 ng BSA/slot; 3: 600 ng BSA/slot; 4: 900 ng BSA/slot; 5–7: E1-3 from mock purification; 8–10: E1-3 from PfGAP50-ERH repeat 1; 11–13: E1-3 from PfGAP50-ERH repeat 2; 14–16: E1-3 from PfGAP50-ERH repeat 3; 17–19: E1-3 from PfGAP50-cTPH repeat 1; 20–22: E1-3 from PfGAP50-cTPH repeat 2; 23–25: E1-3 from PfGAP50-cTPH repeat 3. (PDF 156 kb) [file 12896_2015_225_MOESM1_ESM.pdf]

Additional file 1

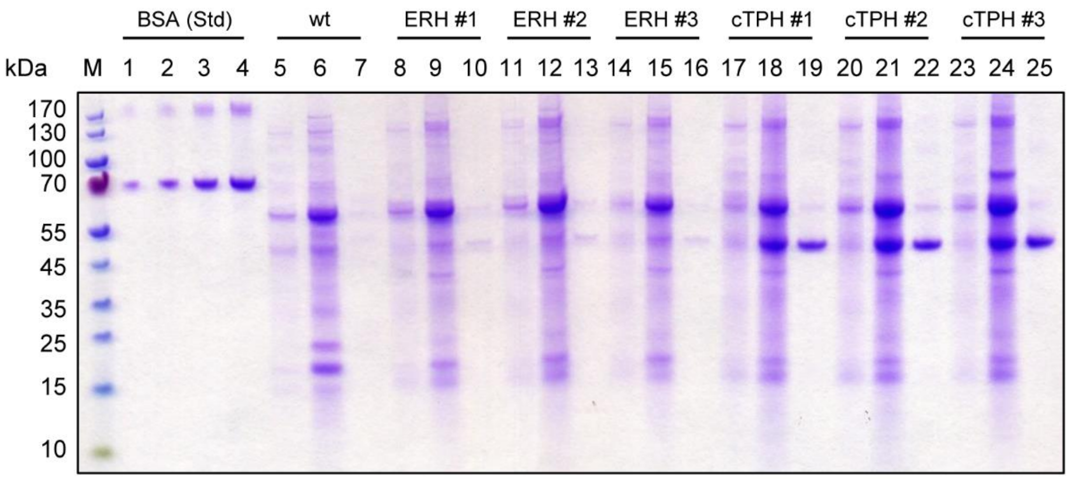

Addtional file 1: SDS-PAGE based quantification (densitometric against a BSA standard) of *Pf*GAP50 variants in IMAC elution fractions (three independent replicate each). Mock purification of wt *N. benthamiana* extract was included. Proteins were eluted from the IMAC column using three elution steps (elution 1 (E1): 10 mM imidazole; elution 2 (E2): 100 mM imidazole and elution 3 (E3): 250 mM imidazole). 6 µl of each sample was loaded under reducing conditions. 1: 150 ng BSA/slot; 2: 300 ng BSA/slot; 3: 600 ng BSA/slot; 4: 900 ng BSA/slot; 5-7: E1-3 from mock purification; 8-10: E1-3 from *Pf*GAP50-ERH repeat 1; 11-13: E1-3 from *Pf*GAP50-ERH repeat 2; 14-16: E1-3 from *Pf*GAP50-ERH repeat 3; 17-19: E1-3 from *Pf*GAP50-cTPH repeat 1; 20-22: E1-3 from *Pf*GAP50-cTPH repeat 2; 23-25: E1-3 from *Pf*GAP50-cTPH repeat 3.
